# Guarding against excess: a frequently ignored ethical principle in medical education research

**DOI:** 10.15694/mep.2018.0000152.1

**Published:** 2018-07-24

**Authors:** Ken Masters

**Affiliations:** 1 Sultan Qaboos University

**Keywords:** Ethics, Ethics, Research, Research, Medical Informatics, Medical Informatics Ethics, Surveys, Questionnaires, Demographics, Demography

## Abstract

This article was migrated. The article was marked as recommended.

In the 21 ^st^ century, medical ethics has been combined with informatics ethics to form medical informatics ethics, and medical education researchers need to be aware of medical informatics ethics principles. An important medical informatics ethics principle is *guarding against excess*: gathering only those data that are required, and using all the data that have been gathered. An example of excessive data gathering can be found in the gathering of demographic data: it is considered *excessive* when these data are gathered for no explicit reason, used minimally in the Results, and mostly ignored in the Discussion. This paper details the problem of excessive data gathering, and then outlines five steps requiring the cooperation of institutional ethics review boards, researchers, journal editors and journal reviewers that should be followed in order to guard against excess in medical education research.

## Introduction: Medical Informatics Ethics

Medical practitioners and researchers are well aware of medical ethics principles.  Much of our guidance in medical research practice stems from the Medical Cases in the Nuremberg Trials ( Nuernberg Military Tribunals, 1949a, 1949b), the resultant rulings ( Nuernberg Military Tribunals, 1949b) that led to the Nuremberg Code ( Anonymous, 1947), and the Declaration of Helsinki. ( WMA, 2008)

The digital world, however, has its own set of ethical principles stemming from informatics and information science, and these are well-entrenched as informatics ethics ( Severson, 1997) and even informatics laws. ( European Parliament, 1995)

When medical ethics and informatics ethics combine, the result is medical informatics ethics.  This is not the place to go into detail about medical informatics ethics, although there are some useful introductions to the topic. ( de Lusignan *et al.*, 2007; Jankowski and van Selm, 2007; Goodman *et al.*, 2013; Masters, 2018)  For the purposes of this paper, it is enough to note that medical informatics ethics governs the ethical use of medically-related data captured or stored electronically.  Because all medical and medical education practitioners and researchers work with such data, they need to be cognisant of medical informatics ethics.

## Guard against excess

Although the recent General Data Protection Regulation (GDPR) alerted millions of Internet users to the complexities of data-gathering and data-storing, the European Union has been concerned with these problems for more than 20 years.  For example, an important informatics ethics principle is explained clearly in article 25 of Directive 95/46/EC of the European Parliament and of the Council of 24 October 1995 on the protection of individuals with regard to the processing of personal data and on the free movement of such data:

*Whereas any processing of personal data must be lawful and fair to the individuals concerned; whereas, in particular, the data must be adequate, relevant and not excessive in relation to the purposes for which they are processed; whereas such purposes must be explicit and legitimate and must be determined at the time of collection of the data; whereas the purposes of processing further to collection shall not be incompatible with the purposes as they were originally specified;[my emphasis]* ( European Parliament, 1995)

In short, when gathering personal data, one must *guard against excess*,be clear about exactly why all data are being gathered, and not gather personal data because they *might* be interesting, or *might* be useful, or just because everyone else gathers those data.  When gathering data, each item must have a correctly-justified reason, and must be used according to those reasons.

## Implications for Medical Education Research

### Medical education surveys

A large amount of medical education research relies on surveys of humans, whether students, residents, teachers or patients.  In order to guard against excess in data-gathering, researchers need to ask themselves some pertinent questions, such as: How much of the information that we gather do we actually need?  How much is collected justifiably?  How much is actually used?  If we do not use it, why did we gather it?  (Any medical researcher will note parallels from medical ethics: do not conduct tests unless the data are actually required).

To demonstrate this principle in medical education research, we look at the example of researchers’ gathering research-subject demographic data.

### Demographic data

As a standard in student surveys, medical education researchers collect data regarding gender and age.  This is performed routinely – I doubt that any reader can recall medical education research on students that did not gather demographic information.

But why?  Unless the literature behind our research indicates that there are statistically-significant differences to be expected between the genders or the age groups, what is the reason for our collecting these data?  Just for interest?  Just to see, *maybe*, if there is something?  Or, perhaps, and more likely, just because every other questionnaire we have seen contains those questions?  Do we really begin every survey form with our standard three or four demographic questions because, well, that is what we have always done, and everybody else does it?   These are not valid justifications to collect information.

Yes, there may be times when there is to be an exploration into data areas that may be unsupported, or only hinted at, by the literature.  In that case, this needs to be stated clearly as part of the Research Aims, detailed in the Methodology, reported on properly in the Results, and discussed comprehensively in the Discussion.

At other times, researchers may want these data in order to ensure that the sample is representative of a wider population.  Again, however, if that is the case, then those researchers need to perform an explicit and statistically valid comparison with that wider population.  A general eye-balling is not a valid comparison, just as it would not be valid for any other data.

And yet we see many papers in which these data are gathered, referred to briefly in the text, with perhaps a simple table, and then forgotten.  A typical example would be a single, isolated line saying something like “76 (51%) of the respondents were female, and 74 (49%) were male” with no further reference to this information, or why it is important for the reader to know this, why it was gathered in the first place, and then it is never referred to elsewhere in the paper.

I shall not embarrass authors by pointing to example papers, but one does not have to look far to see these papers.  Readers can perform an experiment of the next five medical education papers involving surveys that they read: look at the demographic data and ask yourself about the use of those data in the paper, apart from a simple listing.

This is poor scientific work.  We know this, because, if we were performing clinical research, it would be unconscionable to perform tests on research subjects, gather data from those tests, and then not refer to those data in our paper in any meaningful way.   If our Results do not report on these data in any meaningful way (apart from simply noting a few percentages), and we do not refer to these data in the Discussion, then it means that the data were gathered for no apparent reason.  Simply listing these data without any further discussion is of no value at all.  To justify gathering the demographic data, we need to examine those data properly (just as we would examine any other data), and, if we *do* find significant information, then we need to address this.  Otherwise we have simply gathered excess data, and we need to guard against doing so.

This paper has used only demographic data as an example, because demographic data are common to all surveys.  Guarding against excess, however, does not apply only to the demographic data, but to *all* the questions.  To guard against excess, *every* question must have a reason behind it, supported either by the literature or some other acceptable research support, and then must be used meaningfully in the paper.

## Steps to be taken to guard against excessive data gathering

How do we guard against excessive data collection?

First, when research proposals are submitted to ethical review boards, the reason for each question item should be either obvious or explained properly in the proposal.  If this is not clear, then ethical reviews boards need to query the items.

Second, in the submitted manuscript, there should be reporting of any and all data (including demographic data).  Simply listing the gender (or age, or year of study, etc.) and the n of each sub-group tells us nothing about *why* that information was gathered, and what the significance of that information is.  The authors should at least perform statistical tests to see if these demographic groupings matter, and then discuss these results in light of the current literature, just as they do with any other results.  (For instance, if they have been gathered as independent variables, or as possible predictors of other variables’ values, then they must be examined and tested as such).

Third, it might be a good idea for the journal and the reviewers to ask to see the full questionnaire.  If data were gathered on any topic, and not reported on in the paper, then the researchers should be asked to justify why that information was required in the first place.  And “Just for interest” or “Because we always do that” are not justifications.  (A query like this from the journal might also reduce the practice of thin-slicing or salami-slicing of data in medical education publications.)

Fourth, given that journal editors and reviewers might expect, and wish, to see demographic data (simply because, well, that is what *everyone* does), authors should clearly state why some expected data were *not* gathered – like every other action in the Methodology, it should be supported by reference to the literature.

Fifth, journal editors and reviewers should not expect to see data unless there is good reason to have gathered them.  If, for example, a reviewer queries why the researcher did not gather the students’ genders, the response should be simply that the literature does not indicate that this is a significant factor, and that the gender has as much relevance to the topic as the students’ hair length or eye colour.

By following these steps, we can guard against excess in medical education research data gathering.

## Conclusion

In a digital world where data are so easily gathered and processed, medical education researchers should ensure that they follow medical informatics ethics principles.  Among these is guarding against excess when gathering data.  Researchers should gather only what they need, and should use what they have gathered.  By being cognisant of the issues and performing simple checks, medical education researchers, editors and reviewers can ensure that proper safeguards are put in place.  Above all, they should bear in mind that “Just for interest” or “Because we always do that” are not justifications for data-gathering.

## Take Home Messages

• Medical education researchers need to follow medical informatics ethics principles.
• An important principle is guarding against excess when gathering data: gathering only what are needed, and using all that have been gathered.
• Frequently, this principle is ignored in medical education research, and is best demonstrated by the gathering and inadequate use of research-subject demographic data.
• Guarding against excess applies to all questions, and requires the cooperation of institutional ethics review boards, researchers, journal editors and journal reviewers.

## Notes On Contributors

Dr. Ken Masters, PhD, HDE, FDE, Assistant Professor of Medical Informatics, Sultan Qaboos University, Oman, has been involved in medical education for over a decade. His publications consider educational theory, technologies, strategies, and softer areas, such as ethics. He teaches medical informatics ethics as part of a medical informatics course.

## Declarations

The author advises AMEE on aspects of AMEE MedEdPublish, and is an Associate Editor of AMEE MedEdPublish. This paper has been submitted through normal channels, and he has played no role in the decision by AMEE MedEdPublish to publish this paper.

## References

[ref1] Anonymous (1947) The Nuremberg Code, History. NIH. Available at: https://history.nih.gov/research/downloads/nuremberg.pdf( Accessed: 9 July 2018).

[ref2] European Parliament (1995) Directive 95/46/EC of the European Parliament and of the Council of 24 October 1995. Brussels.

[ref3] Goodman K. W. Adams S. Berner E. S. Embi P. J. (2013) AMIA’s Code of Professional and Ethical Conduct. Journal of the American Medical Informatics Association. 20(1), pp.141–143. https://doi.org/10.1136/amiajnl-2012-001035 22733977 10.1136/amiajnl-2012-001035 PMC3555329

[ref4] Jankowski N. and van Selm M. (2007) Research ethics in a virtual world. Guidelines and illustrations.in Carpentier N. Pruulmann-Vengerfeldt P. Nordenstreng K. Hartmann M. (eds) Media technologies and democracy in an enlarged Europe. Tartu: Tartu University Press.

[ref5] de Lusignan S. Chan T. Theadom A. and Dhoul N. (2007) The roles of policy and professionalism in the protection of processed clinical data: A literature review. International Journal of Medical Informatics. 76(SUPPL. 1), pp.261–266. 10.1016/j.ijmedinf.2005.11.003 16406791

[ref6] Masters K. (2018) Health Informatics Ethics.in Hoyt R. E. and Hersch W. R. (eds) Health Informatics: Practical Guide. 7th edn. Pensacola, Florida: Informatics Education, pp.233–251.

[ref7] Nuernberg Military Tribunals (1949a) Trials of war criminals before the Nuernberg Military Tribunals under Control Council Law No. 10, Volume I: ‘The Medical Case. Washington, DC: US Government Printing Office.

[ref8] Nuernberg Military Tribunals (1949b) Trials of war criminals before the Nuernberg Military Tribunals under Control Council Law No. 10, Volume II: ‘The Medical Case’ and ‘The Milch Case. Washington, DC: US Government Printing Office.

[ref9] Severson R. (1997) The Principles of Information Ethics. New York: M.E. Sharp.

[ref10] WMA (2008) Declaration of Helsinki. Available at: https://www.wma.net/wp-content/uploads/2016/11/DoH-Oct2008.pdf( Accessed: 9 July 2018).

