# Supplementary material for: Guarding against excess: a frequently ignored ethical principle in medical education research
Source: MedEdPublish (2016). 2018 Jul 24;7:152. [Version 1] doi: 10.15694/mep.2018.0000152.1 (PMC10701839; doi:10.15694/mep.2018.0000152.1)
Supplement: Peer review history

# Reviewer report: Reviewer response for version 1

**DOI:** 10.21956/mep.19628.r29213

**Published:** 2018-12-05

**Authors:** Richard Hays

**Affiliations:** 1 James Cook University

This review has been migrated. The reviewer awarded 4 stars out of 5 Thank you for this interesting contribution to the discussion on research ethics and data collection. My interest comes from my roles as a researcher and an editor. The ubiquity of data collection and the frequently reported data leaks are part of life now, but just because data collection and storage is so easy now, what should be collected for what purpose? As a researcher, I can remember losing several debates on what to include/exclude in questionnaire design. I have always favoured a minimalist approach - ask only questions that are related to the research question or issue. This approach also results in shorter questionnaires that might achieve a higher response rate. However, the pressure to add questions 'just in case' there is a correlation is strong and often holds sway. As a consumer of research, my least favourite reports are those that report multiple correlations that are simple associations, without cause and effect relationships. This form of research is common in public health survey research, showing that certain foods, alcohol, additives etc may be associated with either shorter or longer lives etc. In essence, the results are often not particularly meaningful or helpful . As Editor of MedEdPublish, which invites reviews after publication, our position is not to make prior judgements about such issues, but comments on ethics and data collection are very welcome in both panelist and reader reviews.

Reviewer Expertise:

NA

No decision status is available

# Reviewer report: Reviewer response for version 1

**DOI:** 10.21956/mep.19628.r29215

**Published:** 2018-07-26

**Authors:** Faraz Khurshid

**Affiliations:** 1 University of Buraimi, College of Health Sciences

This review has been migrated. The reviewer awarded 4 stars out of 5 An illuminating chunk of information. Excessive data gathering is an inevitable problem that can be an alarming threat to the quality of literary work contributed by the league of scientific and research communities. The author provided ways to address this problem wisely. The suggestions are reasonable and can help to improve the guidelines of research and ethics linked to scientific endeavours.

Reviewer Expertise:

NA

No decision status is available

# Reviewer report: Reviewer response for version 1

**DOI:** 10.21956/mep.19628.r29216

**Published:** 2018-07-24

**Authors:** P Ravi Shankar

**Affiliations:** 1 American International Medical University

This review has been migrated. The reviewer awarded 4 stars out of 5 I would like to thank the author of enlightening me about excessive data collection in medical education research. The issue of excessive collection of demographic data which is not used in the analysis of the results and in the discussion section is very well described. I have been educated about this issue and will keep it in mind while conducting future research studies. I also plan to use this principle in approving research projects and in reviewing research articles.

Reviewer Expertise:

NA

No decision status is available

# Reviewer report: Reviewer response for version 1

**DOI:** 10.21956/mep.19628.r29214

**Published:** 2018-07-24

**Authors:** Nandalal Gunaratne

**Affiliations:** 1 Faculty of Medicine, Wayamba University of Sri Lanka

This review has been migrated. The reviewer awarded 3 stars out of 5 Thank you. This is something not really looked into at the ethical review committee level. Perhaps this first step, with a good look at the questionnaire, will bring in greater discipline in data collection.

Reviewer Expertise:

NA

No decision status is available
